# MicroRNA-181a Is Involved in Methamphetamine Addiction Through the ERAD Pathway

**DOI:** 10.3389/fnmol.2021.667725

**Published:** 2021-05-07

**Authors:** Yujing Wang, Tao Wei, Wei Zhao, Zixuan Ren, Yan Wang, Yiding Zhou, Xun Song, Ruidong Zhou, Xiaochu Zhang, Dongliang Jiao

**Affiliations:** ^1^School of Mental Health, Bengbu Medical College, Bengbu, China; ^2^Chinese Academy of Sciences (CAS) Key Laboratory of Brain Function and Disease and School of Life Sciences, University of Science and Technology of China, Hefei, China

**Keywords:** methamphetamine, miR-181a, endoplasmic reticulum-associated protein degradation, GABAAα1, addiction

## Abstract

The regulation of microRNA (miRNA) is closely related to methamphetamine (METH) addiction. Past studies have reported that miR-181a is associated with METH addiction, but the mechanism pathways remain elusive. On the basis of our past studies, which reported the endoplasmic reticulum-associated protein degradation (ERAD) mediated ubiquitin protein degradation of GABAAα1, which was involved in METH addiction. The present study, using qRT-PCR and bioinformatics analysis, further revealed that miR-181a may be indirectly responsible for the METH addiction and downregulation of GABAAα1 through the regulation of ERAD.

## Introduction

Drug abuse has been widely recognized as a debilitating chronic disease of the brain, and it is one of the most dangerous diseases to human physical and mental health in modern society.

Methamphetamine (METH), one of the commonly used drugs, is addictive and neurotoxic. Long-term use of methamphetamine can lead to addiction, causing varying degrees of physical and psychological harm to the addicts (Anna and Patrick, 2017), and methamphetamine (MA) abuse is considered a serious global public health problem. Despite the severity of the consequences of drug abuse, there is still no fully effective treatment for methamphetamine addiction. Therefore, research into the mechanisms of methamphetamine addiction is critical.

Traditionally, the main mechanism of ATS addiction is the increased activity of the midlimbic dopamine system. However, the neurobiology of METH is more complex than the traditional view of it as a monoaminergic modulator. It may include oxidative stress, excitatory neurotoxicity, endoplasmic reticulum stress, and neuroinflammation (Paulus and Stewart, 2020). More complex regulatory mechanisms may also be involved in the addictive process of METH.

MicroRNAs (miRNAs) are small (21–25-nucleotides long), non-coding RNA molecules that are responsible for the suppression of protein expression by targeting the mRNA of protein-coding genes through translational repression (Reuben et al., 2012). MicroRNAs act as regulators of gene expression and play a key regulatory role in different drug addiction. Some studies showed that miRNAs have been implicated as mediating the effects of cocaine (Hollander et al., 2010; Bali and Kenny, 2013), alcohol (Torres et al., 2018; Kyzar et al., 2019), nicotine (Qin et al., 2017; Du et al., 2019), and several other classes of drugs (Rodríguez, 2012; Pinson and Miranda, 2019; Zhang et al., 2019). A growing body of evidence suggests that miRNAs and their processing machinery is involved in in METH addiction (Zhu et al., 2015a; Sim et al., 2017). Previous studies have reported the involvement of miR-181a in METH addiction (Reuben et al., 2012; Kai et al., 2016; Zhao et al., 2016). However, the mechanisms involved need to be further elucidated. Our earlier study revealed that the GABAAα1 expression was downregulated in the dorsal striatum (Dstr) of METH-addicted rats, which was caused by protein ubiquitin degradation and mediated by endoplasmic reticulum-associated protein degradation (ERAD) (Jiao et al., 2016, 2017). Taking into account the role of miR-181a in direct regulation of GABAAα1 (Zhao et al., 2012; Sengupta et al., 2013), it was assumed that miR-181a plays some role in the downregulation of GABAAα1. On the basis of the experimental study and bioinformatics analysis, it was found that miR-181a may be involved in the downregulation of GABAAα1 through the regulation of the ERAD pathway and not through direct regulation of the expression of GABAAα1, thus influencing METH addiction.

## Experimental Methods

### Animals and Drug

Sprague–Dawley male rats weighting 220–300 g (aging 50–60 days) were obtained from the Laboratory Animal Center, Bengbu Medical College (Bengbu, Anhui, China). Rats were housed 2–3 per cage and maintained on a 12-h light/dark cycle with access to food and water *ad libitum*. All experimental procedures in this manuscript were in strict accordance with the National Institutes of Health Guide for the Care and Use of Laboratory Animals (NIH Publications No. 80-23, revised 1996) and approved by the Institutional Animal Care and Use Committee of Bengbu Medical College (Bengbu, Anhui, China). METH was provided by China Academy of Military Medical Science.

### Conditioned Place Preference (CPP)

The CPP apparatus (Zhenghua Software and Instruments, Anhui, China) was divided into two equal-sized compartments [40 cm (length) × 40 cm (width) × 60 cm (height)] separated by a removable board (10 × 10 cm), which allowed rats free access to each compartment. Two compartments were distinguished by visual and tactile cues: one was a black and white horizontal striped wall with an iron wire floor, whereas the other was a black and white vertical striped wall with a steel bar floor. These distinctive tactile and visual stimuli served as the conditioning.

CPP model follows the experimental reported in our previous article (Jiao et al., 2016, 2017). The place conditioning procedure used in this experiment included four phases: habituation, preconditioning, conditioning, and testing. In the habituation phase, the rats were allowed to freely explore the entire apparatus for 30 min. In the preconditioning phase, the rats were allowed to freely explore the entire apparatus for 15 min. The time spent in each compartment was recorded in the preconditioning phase, and rats showing a strong unconditioned aversion (one compartment >720 s) for either compartment were eliminated from the study. Conditioning occurred over the next 8 days. On the first day of the conditioning phase, the rats were injected with either METH (1 mg/kg, i.p.) or saline (1 ml/kg, i.p.) and then confined to the non-preferred compartment in a counterbalanced manner for 45 min. This compartment will be referred to as the “drug treatment-paired compartment.” On the second day, the rats were injected with saline (1 ml/kg, i.p.) and then confined to the opposite compartment from the first day for 45 min. This procedure was repeated four times in the conditioning phase. The testing phase occurred 24 h after the conditioning trial, and all rats were allowed to freely explore the entire apparatus for 15 min; the amount of time spent in each compartment was recorded. The CPP score represents the time in the drug treatment-paired compartment during the testing phase minus the time in that compartment during the preconditioning phase.

### Quantitative Reverse Transcriptase PCR (qRT-PCR)

Rats were sacrificed and the brains were removed immediately after CPP conditioning. The brain was dissected into coronal slices (1 mm thick) using a rat brain slicer (Braintree Scientific), and the dorsal striatum was punched by a blunt end, 17-gauge syringe needle. Total RNA was isolated from rat Dstr using a commercially available kit (RNeasy Plus Mini Kit, Qiagen). QRT-PCR was performed on a ABI7500 Real-Time PCR system (ABI) using SYBRr Premix Ex TaqTM kit (TaKaRa Bio Group, Japan). A typical reaction of a total volume of 20 μl consisted of 2 μl Template DNA, 10 μl 2 × SYBR Green Reaction Mix, 0.4 μl PCR Forward Primer (10 mM), 0.4 μl PCR Reverse Primer (10 μM), 0.4 μl ROX Reference Dye II and 6.8 μl DEPC treated water. PCR amplification was done with an initial incubation at 95°C for 30 s, and then followed by 40 cycles of 95°C for 5 s, 60°C for 34 s, and final melting curve from 95°C for 5 s, 60°C for 60 s. Primer specificity was confirmed by melting curve analysis.

Primers utilized were as follows: rno-miR-181a-5p, MIMAT0000858: 5′AACAUUCAACGCUGUCGGUGAGU; rno-miR-181b-5p, MIMAT0000859: 5′AACAUUCAUUGCU GUCGGUGGGU; rno-miR-181c-5p, MIMAT0000857: 5′AAC AUUCAACCUGUCGGUGAGU; rno-miR-181d-5p, MIMA T0005299: 5′AACAUUCAUUGUUGUCGGUGGGU; Hs_RNU 6-2_11, GeneGlobe Id: MS00033740. Above primers were purchased from Qiagen Company.

UBE2D3: Fdw-5′CTATGGCGCTGAAACGGATT, Rev-5′G GGCTGTCATTAGGTCCCAT; RNF169: Fdw-5′TCCATTCCA GCAAGGAGAGG, Rev-5′GGAATGGCAGGTTTCCAGTG; FB XO33: Fdw-5′CTCAGCATCCGGAACAACAG, Rev-5′ATAA ACCCACAGGACAGCCA; RNF145: Fdw-5′TCTCCAGGTTC TGGGAACAC, Rev-5′GCGGTAAGTGCCATTCACAT; RAD 23B: Fdw-5′TCTGAACCTGCACCTACTGG, Rev-5′AGACTG ACCTGTCACAAGGG; NEURL1B: Fdw-5′CTGGTCACACG ACCTGGATA, Rev-5′TGGTTCGCCATCATTGACAC; PCN P: Fdw-5′GCGATCAGCTGAAGACGAAG, Rev-5′; KLHL 15: Fdw-5′ATAGACGACGGTGGAGACAC, Rev-5′CAACAA GGGTTGCTGGTGAA; KLHL5: Fdw-5′GCTCCCACATCCAA CTTGAC, Rev-5′CACTGCAGTCCACATGTCTG; TULP4: F dw-5′GAGCATGGACCTCTGCTTTG, Rev-5′GTGGCCAACC ATCCTTCTTC; RNF34: Fdw-5′AAGGAAATCCTGGCTCG GAA, Rev-5′TCCAGCAGGACACAGTCAAT; KLHL2: Fdw-5′GAGTACTTGGTGCAGAGGGT, Rev-5′AGGTTCATGGGTG TCCTCAG; CAND1: Fdw-5′TGCCAGAAGCTCAGTGGTTA, Rev-5′CAGTGACGGCTTGTTATGGG; HSP90B1: Fdw-5′A CTGCATTCAGGCTCTTCCT, Rev-5′TCTCTGTTGCTTCC CGACTT; DERL1: Fdw-5′GCCATGGATATGCAGTTGCT, Rev-5′TTGATGACCGAGCCTCCAAT; HSPA5: Fdw-5′GGTGGGCAAACCAAGACATT, Rev-5′TCAGTCCAGCAA TAGTGCCA; GAPDH:Fwd-AACTTTGGCATTGTGGAAGG, REV-5′ACACATTGGGGGTAGGAACA.

Above primers were purchased from Genewiz in China.

### Bioinformatic Analysis

The details regarding the base sequence and species conservation of miR-181a-5p were retrieved from the online database miRbase^1^. Using Venn plot, the target gene of miR-181a-5p was screened by the intersection of TargetScan^2^, miRDB^3^, and miRanda^4^ online databases. Functional enrichment analysis was performed using the DAVID (Database for Annotation, Visualization, and lntegrated Discovery)^5^ online database, which included gene function GO (Gene Ontology) analysis (such as biological process, cellular component, molecular function) and KEGG (Kyoto Encyclopedia of Genes and Genomes) pathway enrichment analysis. STRING (Search Tool for the Retrieval of Interaction Gene)^6^, an online software, was used to analyze the interaction between target gene-encoded proteins and to visualize the protein interaction networks. RNA high-throughput sequencing data of animal brain tissues treated with METH were retrieved from the EMBL-EBI (European Molecular Biology Laboratory’s European Bioinformatics Institute)^7^ (Zhu et al., 2015b) online database, and cluster analysis was performed where *P* < 0.05 was considered to be statistically significant.

### qRT-PCR Statistical Analysis

U6 or GAPDH was used as an endogenous control for the qRT-PCR. Fold changes in expression were calculated with a log 2 transform. Independent-sample *t*-tests were used to test for significant differences (SPSS v23.0, SPSS Inc., United States). Differences in the values with *p* < 0.05 were considered to be statistically significant. These results were presented as mean ± standard deviation (SD).

## Results

### Decrease in miR-181a-5p and miR-181b-5p Expressions in METH-Induced CPP Rat

**FIGURE 1:**
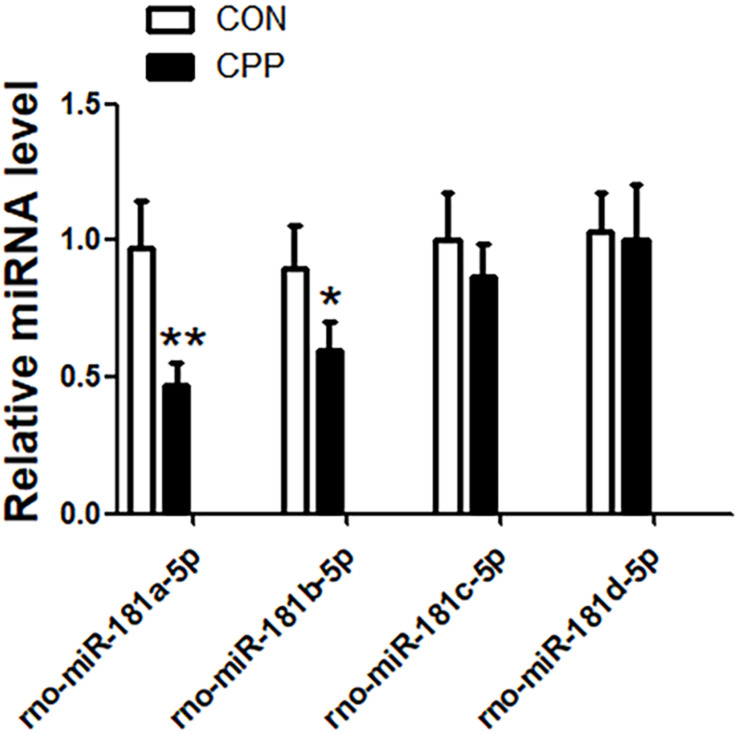
The expression of miR-181a-5p and miR-181b-5p were downregulated in the dorsal striatum of METH-induced CPP rats group, but the expression of miR-181c-5p and miR-181d-5p were not changed. mRNA was determined by qRT-PCR, and the values were normalized to the geometric mean of a control gene U6. Data represent the mean ± SD, *n* = 8 in each group. *P < 0.05 and **P < 0.01 compared with control group, two tailed Student’s *t*-test.

The expression levels of miR-181a-5p, miR-181b-5p, miR-181c-5p, and miR-181d-5p in the dorsal striatum of METH-induced CPP rats were investigated (Figure 1). The results indicated that the expression of miR-181a-5p (*n* = 8, ^∗∗^*p* < 0.01) and miR-181b-5p (*n* = 8, ^∗^*p* < 0.05) were downregulated in the METH-induced CPP group when compared to the control group, while the expression of miR-181c-5p and miR-181d-5p did not show any significant change (*n* = 8, ^∗^*P* > 0.05). Keeping in mind the findings from several studies (Kai et al., 2016; Zhao et al., 2016) reporting significant decrease in the expression of miR-181a-5p, bioinformatics analysis was performed on miR-181a-5p.

### Conservation Analysis of miR-181a-5p

**FIGURE 2:**
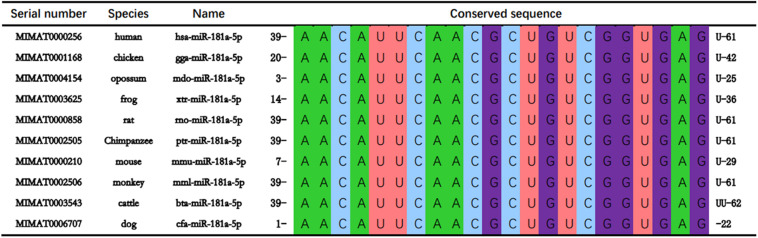
miR-181a-5p are conserved in at least 10 vertebrate species including human (Homo sapiens; hsa), mouse (Mus musculus; mmu), chicken (Gallus gallus; gga), opossum (onodelphis domestica; mdo), frog (Xenopus tropicalis; xtr), rat (Rattus norvegicus; rno), Chimpanzee (Pan troglodytes; ptr), monkey (Macaca mulatta; mml), cattle (Bos taurus; bta), and dog (Canis familiaris; cfa).

The mature sequences of miR-181a-5p of 10 species including human (hsa), rat (rno), chicken (gga), and frog (xtr) were retrieved from online data base, miRbase^1^. The mature sequences of each of the gene family of miR-181a-5p gene cluster in different species were compared and revealed that the sequence of miR-181a-5p gene cluster AACAUUCAACGCUGUCGGUGAG was highly conserved in the vertebrates (Figure 2).

### Establishment of miR-181a-5p Target Gene Set

**FIGURE 3:**
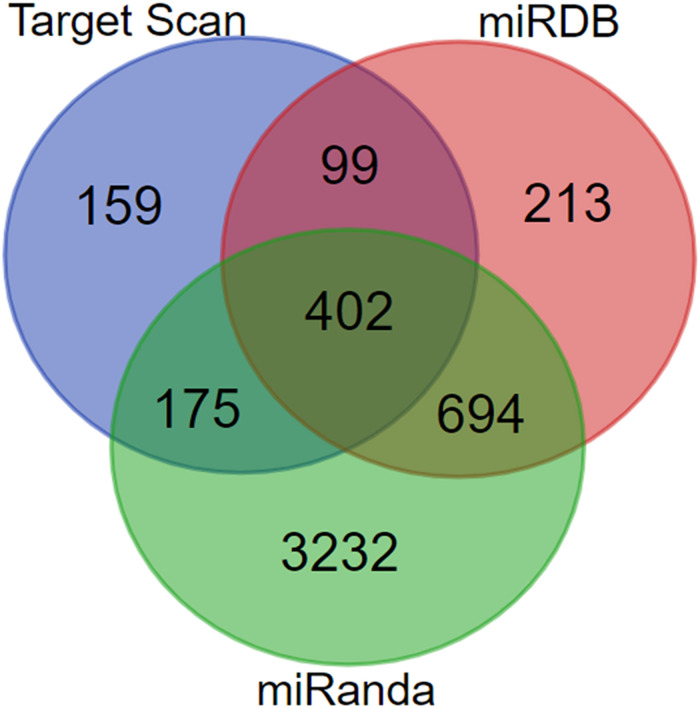
Construction of miR-181a-5p target gene set.

The number of target genes predicted by the three online databases (TargetScan, miRDB, and miRanda) were 835, 1408, and 4503, respectively. After the target genes were screened by the intersection, a total of 402 unreplicated target genes were obtained as the total gene set for subsequent analyses (Figure 3).

### GO and KEGG Enrichment Analyses of miR-181a-5p Target Gene

**FIGURE 4:**
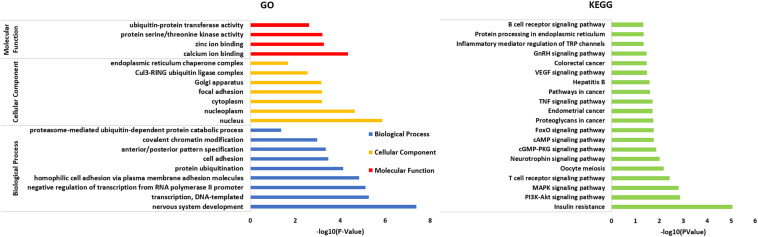
All 402 target genes analyzed by DAVID software and the results of GO analysis and KEGG analysis (*P* < 0.05) shown in the figure above. The results of GO analysis were classified into three functional groups, including biological process (BP), cellular component (CC) and molecular function (MF). On the basis of GO item classification, the KEGG analysis of the target gene set was mainly enriched in multiple biological pathways (*P* < 0.05), for example, MAPK signaling pathway, cAMP signaling pathway, TNF signaling pathway and the specific molecular pathway of the endoplasmic reticulum chaperone complex involved in the ubiquitin-dependent protein catabolic process.

A total of 402 target genes were analyzed by the DAVID software, and the results of GO analysis and KEGG analysis have been presented in Figure 4. Among them, 36 genes related to the endoplasmic reticulum and ubiquitin degradation are shown in Table 1. Table 1 indicated that (1) for biological processes (BP), twenty-two genes (KLHL29, RNF34, UBE2B, KLHL15, CUL3, MYCBP2, UBE2D3, TULP4, MED8, KLHL2, TNFAIP1, FBXO33, MID2, BACH2, PCNP, NEURL1B, RNF169, CAND1, KLHL5, TRIM2, RNF182, BIRC6) are particularly enriched in protein ubiquitination and 10 genes (ABTB2, PCNP, RNF145, RNF34, UBE2B, CUL3, BTBD3, UBE2D3, TNFAIP1, RAD23B) are particularly enriched in proteasome-mediated ubiquitin-dependent protein catabolic process. (2) For cell component (CC), seven genes (KLHL29, KLHL5, KLHL15, CUL3, KLHL2, TNFAIP1, BACH2) are particularly enriched in Cul3-RING ubiquitin ligase complex and three genes (HSPA5, HYOU1, HSP90B1) are particularly enriched in endoplasmic reticulum chaperone complex. (3) For molecular function (MF), seventeen genes (KLHL29, TRIM71, RNF34, UBE2B, UBE3C, KLHL15, CUL3, UBE2D3, KLHL2, CBLB, TNFAIP1, BACH2, KLHL5, HECW2, TRIM2, RNF182, BIRC6) are particularly enriched in ubiquitin-protein transferase activity. (4) KEGG analysis results were shown in Table 1, which demonstrated that eight genes (HSPA5, SEL1L, UBE2D3, DERL1, HYOU1, SEC24C, RAD23B, HSP90B1) were particularly enriched in protein processing in endoplasmic reticulum.

**TABLE 1:** 36 genes are particularly enriched in protein ubiquitination and endoplasmic reticulum chaperone complex involved in ubiquitin-dependent protein catabolic process.

Category	Term	Count	PValue	Genes	Benjamini
BP	GO:0016567∼protein ubiquitination	22	7.39E-05	KLHL29, RNF34, UBE2B, KLHL15, CUL3, MYCBP2, UBE2D3, TULP4, MED8, KLHL2, TNFAIP1, FBXO33, MID2, BACH2, PCNP, NEURL1B, RNF169, CAND1, KLHL5, TRIM2, RNF182, BIRC6	0.028745
BP	GO:0043161∼proteasome-mediated ubiquitin-dependent protein catabolic process	10	0.041985	ABTB2, PCNP, RNF145, RNF34, UBE2B, CUL3, BTBD3, UBE2D3, TNFAIP1, RAD23B	1
CC	GO:0031463∼Cul3-RING ubiquitin ligase complex	7	0.00281	KLHL29, KLHL5, KLHL15, CUL3, KLHL2, TNFAIP1, BACH2	0.134878
CC	GO:0034663∼endoplasmic reticulum chaperone complex	3	0.021374	HSPA5, HYOU1, HSP90B1	0.598462
MF	GO:0004842∼ubiquitin-protein transferase activity	17	0.002424	KLHL29, TRIM71, RNF34, UBE2B, UBE3C, KLHL15, CUL3, UBE2D3, KLHL2, CBLB, TNFAIP1, BACH2, KLHL5, HECW2, TRIM2, RNF182, BIRC6	0.20809
KEGG_PATHWAY	hsa04141:Protein processing in endoplasmic reticulum	8	0.046659	HSPA5, SEL1L, UBE2D3, DERL1, HYOU1, SEC24C, RAD23B, HSP90B1	0.286044

**FIGURE 5:**
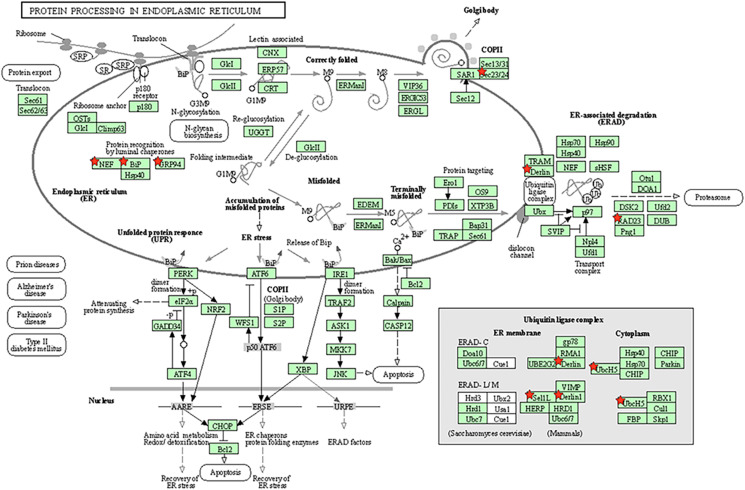
Molecular pathway of endoplasmic reticulum chaperone complex involved in ubiquitin-dependent protein catabolic process. Genes marked the little red pentagram were genes regulated by miR-181a. Figure was cited from DAVID online database.

The specific molecular pathway of the endoplasmic reticulum chaperone complex involved in the ubiquitin-dependent protein catabolic process has been shown in Figure 5.

### Interaction Analysis of miR-181a Target Gene With SYVN1 and GABAAα1

**FIGURE 6:**
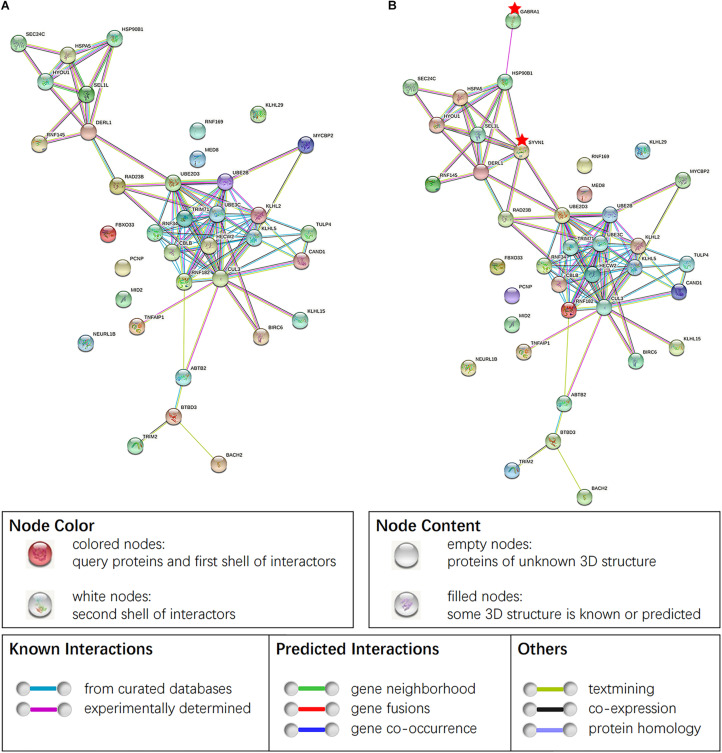
**(A)** PPI of 36 target genes of miR-181a-5p. **(B)** PPI obtained by taking SYVN1 and GABAAα1 together with 36 genes in STING analysis. Network nodes represent proteins, proteins splice isoforms or post-translational modifications are collapsed, i.e., each node represents all the proteins produced by a single, protein-coding gene locus. Edges represent protein-protein associations, associations are meant to be specific and meaningful, i.e., proteins jointly contribute to a shared function. Genes marked the little red pentagram were SYVN1 and GABAAα1. Figure was cited from STRING online database.

Thirty-six target genes obtained from GO and KEGG analyses were analyzed by the STRING software for studying the construction of the protein–protein interaction (PPI) network (Figure 6A). The study conducted by the investigators earlier found that SYVN1 was involved in ERAD-mediated ubiquitination of GABAAα1. Therefore, SYVN1, GABAAα1, and 36 other target genes were used for the purpose of analysis by STRING analysis. Figure 6B represents the interaction among these genes, it indicated that the 36 target genes and SYVN1 synergistically were involved in endoplasmic reticulum-related GABAAα1 protein degradation.

### METH Increased the Expression of Some Target Genes Regulated by miR-181a-5p in RNA High-Throughput Sequencing Data

**FIGURE 7:**
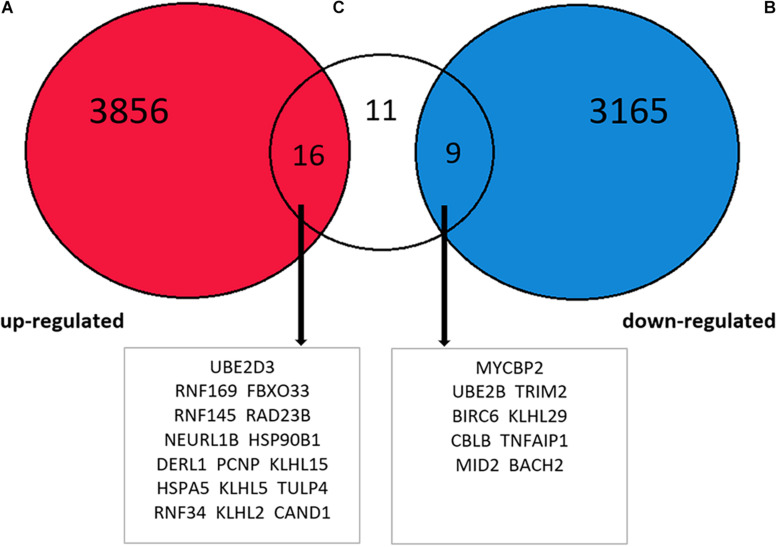
Intersection between EMBL-EBI website METH processing animal brain tissue RNA high-throughput sequencing data analysis results and 36 target genes. The increased expression of 16 target genes, decreased expression of 9 target genes, and 11 target genes without any changes.

To investigate whether the expression of target genes mentioned above increased after METH treatment, RNA high-throughput sequencing data obtained from animals treated with the approximate dose as in this experiment (2 mg/kg) was retrieved from the EMBL-EBI website and cluster analysis was conducted. The level of significance was set at *P* < 0.05, and it was found that the expression levels of 7,046 genes altered after treatment with METH, out of which 3,872 genes exhibited increase in their expression level while 3,174 genes showed a decrease. After screening the results of the intersection of the abovementioned 36 target genes, the results revealed that METH enhanced the expression of 16 target genes, suppressed the expression of 9 target genes, while the expression of 11 target genes remained unchanged (Figure 7), indicating that the expression of some target genes regulated by miR-181a-5p changed on receiving METH treatment. Thus, we demonstrated that the ERAD process was activated after METH treatment.

### The Expression of 16 Target Genes in the Dorsal Striatum of METH-Induced CPP Rats Was Verified by qRT-PCR

**FIGURE 8:**
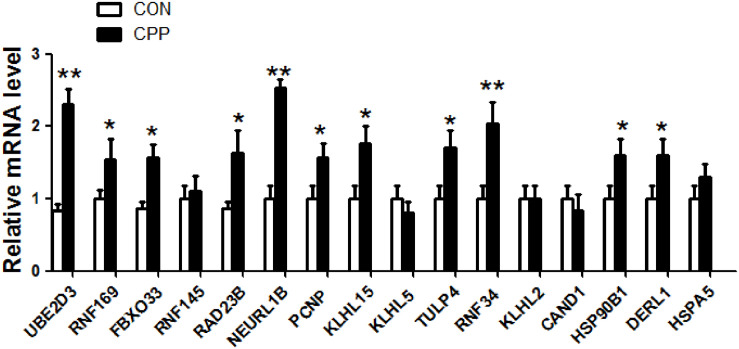
The expression of 16 target genes in the dorsal striatum of METH-induced CPP rats was verified by qRT-PCR. The results showed that mRNA expression of 11 target genes was increased, and 5 target genes had no change. The values were normalized to the geometric mean of a control gene GAPDH (Glyceraldehyde-3-Phosphate Dehydrogenase). Data represent the mean ± SD, *n* = 8 in each group. *P < 0.05 and **P < 0.01 compared with control group, two tailed Student’s *t*-test.

To further verify whether the 16 elevated target genes (UBE2D3, RNF169, FBXO33, RAD23B, NEURL1B, PCNP, TULP4, RNF34, DERL1, HSP90B1, RNF145, KLHL15, KLHL5, CAND1, KLHL2, HSPA5) in Figure 7 are also increased in the dorsal striatum of METH-induced CPP rats. The expression levels of 16 target genes in the dorsal striatum of METH-induced CPP rats were investigated by qRT-PCR. The results showed that the expression levels of 11 target genes, UBE2D3, RNF169, FBXO33, RAD23B, NEURL1B, PCNP, TULP4, KLHL15, RNF34, DERL1, HSP90B1, were upregulated in the METH-induced CPP group when compared to the control group (*n* = 8, *P* < 0.05), while the expression of 5 target genes, RNF145, KLHL5, CAND1, KLHL2, HSPA5, did not change significantly (*n* = 8, *P* > 0.05) (Figure 8).

## Discussion

Our earlier study revealed that the expression of GABAAα1 in the dorsal striatum of METH-addicted rats decreased, which was associated with the protein ubiquitin degradation, mediated by ERAD (Jiao et al., 2017). Past studies indicated miR-181a directly regulates the expression of GABAAα1 (Zhao et al., 2012; Sengupta et al., 2013). Keeping this aspect in mind, we attempted to ascertain the expression levels of miR-181a in the dorsal striatum of METH-addicted rats in order to determine whether the decrease of GABAAα1 expression was regulated by miR-181a. However, it was found out that the expression of miR-181a was downregulated. As an inhibitor of the target gene, miRNA showed the potential to inhibit the translation of mRNA or direct degradation of specific mRNA by pairing with 3′-UTR base of mRNA molecules of a specific target gene. The results of the study revealed that the expression of miR-181a decreased, which is contradictory to the findings from past studies, which reported miR-181a directly regulates the expression of GABAAα1. Studies conducted earlier have also reported that METH downregulates the expression of miR-181a (Kai et al., 2016; Zhao et al., 2016; Sim et al., 2017); this finding is consistent with the results of the present study. The obtained results suggest that miR-181a may not be directly involved in the downregulation of GABAAα1, but probably regulates the expression of GABAAα1 through other pathways.

In this study the results of bioinformatics analysis suggested that the regulation of target genes and molecular pathways by miR-181a involved proteasome-mediated ubiquitin-dependent protein catabolic process and endoplasmic reticulum chaperone complex. Taking into consideration the findings from the past studies, it was found that ubiquitin E3 ligase SYVN1 was involved in the downregulation of GABAAα1 in the dorsal striatum of METH-addicted animals through ERAD (Jiao et al., 2017). It was speculated that miR-181a was possibly related to METH addiction and the downregulation of GABAAα1 through the regulation of ERAD.

GABAAα1 folds and assembles correctly in the endoplasmic reticulum and gets exported to cell membrane for physiological function. Previous studies have found that METH caused dysfunction of the endoplasmic reticulum because of unfolded or misfolded proteins. The proteins get accumulated in the endoplasmic reticulum and are followed by the endoplasmic reticulum stress (Jayanthi et al., 2009; Wongprayoon and Govitrapong, 2017). METH may also induce the unfolded or misfolded GABAAα1. Misfolded GABAAα1 participates in the ubiquitin-degradation process through ERAD and finally gets degraded by proteasome, resulting in the downregulated expression of GABAAα1. This process involves the activation of the ubiquitination system and the endoplasmic reticulum chaperone system.

The results of GO and KEGG analyses by using the DAVID software revealed that 36 target genes and molecular pathways, which were regulated by miR-181a, involved ubiquitin-mediated protein degradation and endoplasmic reticulum chaperone. The results of further analysis conducted using the STRING software provided results that support the interaction of these 36 target genes with SYVN1 in association with GABAAα1. The high-throughput RNA sequencing database of animal brain tissue treated with METH available on the EMBL-EBI website were retrieved for the purpose of cluster analysis. After intersecting the results of cluster analysis with the 36 target genes, it was found that 16 of the target genes regulated by miR-181a experienced upregulation after induction with METH. In order to verify whether the expression levels of 16 elevated target genes in Figure 7 are also increased in the dorsal striatum of METH-induced CPP rats, expression levels of 16 genes in the dorsal striatum of METH-induced CPP rats were detected by qRT-PCR. The results showed that 11 of the 16 target genes were up-regulated. Among the 11 target genes, 9 genes (UBE2D3, RNF169, FBXO33, RAD23B, NEURL1B, PCNP, KLHL15, TULP4, RNF34) were responsible for protein ubiquitination, ubiquitin-protein transferase activity and protein-mediated ubiquitin-dependent protein catalytic process, and 2 genes (DERL1, HSP90B1) were responsible for processing of the proteins in the endoplasmic reticulum. It is further confirmed that METH may activate the ERAD pathway through miR-181 and may induce the ubiquitination degradation of GABAAα1, which is involved in the formation of addiction.

The results of the present study revealed that the expression of miR-181a decreased after receiving methamphetamine treatment. This finding was consistent with the findings of past studies (Kai et al., 2016; Zhao et al., 2016; Sim et al., 2017). However, some studies found that the expression of miR-181a increased in the peripheral blood of animals (Reuben et al., 2012) or cocaine addicts (Viola et al., 2019) treated with other excitatory drugs such as cocaine and amphetamines, which can be attributed to the differential effects of drugs on miRNA production.

In addition, it has been speculated that the downregulated expression of miR-181a in the nervous system may also plays an endogenous protective role. The possible reason is that the enhanced ERAD process helped decrease the accumulation of misfolded proteins and, consequently, decreased endoplasmic reticulum stress and apoptosis (Hwang and Qi, 2018). ERAD can also regulate Alzheimer’s amyloid pathology and memory function by modulating degradation of Aβ protein (Zhu et al., 2017). Previous studies have reported that low-dose METH pre-treatment has a protective effect. For example, studies have found that the early use of low-dose METH pre-treatment could lower the neurological injury score of patients with craniocerebral injury (Duong et al., 2018; El Hayek et al., 2020). Low-dose METH pretreatment improved the cognitive functions of Alzheimer’s patients (Shukla et al., 2019; Shukla and Vincent, 2020) and reduced the neurotoxic effects of high dose of METH (Lu et al., 2019). MiR-181a also plays an essential role in cell proliferation, apoptosis, differentiation, immunity, and tumor progression (Chang et al., 2014). Studies have also found that the high expression of miR-181a inhibits cell growth (Zhang et al., 2013) and promotes cell apoptosis (Zhu et al., 2012). Therefore, the downregulation of miR-181a induced by METH may play a protective role in promoting nerve cell growth and in inhibiting nerve cell apoptosis. This finding calls for further research on miR-181a, which may provide a useful reference for further exploring the protective effects of low-dose METH pre-treatment.

## Conclusion

The results of the bioinformatics analysis revealed that miR-181a can cause ubiquitination of GABAAα1 and induce METH addiction through the regulation of ERAD. Further experimental studies need to be conducted in order to probe deeper into the role of miR-181a in METH addiction. This study provides preliminary findings on the role of miR-181a in drug addiction, which may provide a new intervention target for the treatment of methamphetamine addiction. Further studies need to be conducted to determine the role of miR-181a in neuroprotective effects of low-dose METH treatment.

## Data Availability Statement

The data used to support the findings of this study are available from the corresponding author upon request.

## Ethics Statement

The animal study was reviewed and approved by the Institutional Animal Care and Use Committee of Bengbu Medical College (Bengbu, Anhui, China).

## Author Contributions

YuW and TW wrote the first draft of the manuscript. DJ and WZ provided critical revision of the manuscript for important intellectual content. All authors gave input to the manuscript text, approved the final version of the manuscript, and materially participated in the manuscript preparation.

## Conflict of Interest

The authors declare that the research was conducted in the absence of any commercial or financial relationships that could be construed as a potential conflict of interest.

## Footnotes

1 http://www.mirbase.org/

2 http://www.targetscan.org/vert_72/

3 http://mirdb.org/

4 http://cbio.mskcc.org/microrna_data/miRanda-aug2010.tar.gz

5 https://david.ncifcrf.gov/

6 https://string-db.org/

7 https://www.ebi.ac.uk/arrayexpress/experiments/E-MTAB-2843/samples/
